# Amino Functionalization of Reduced Graphene Oxide/Tungsten Disulfide Hybrids and Their Bismaleimide Composites with Enhanced Mechanical Properties

**DOI:** 10.3390/polym10111199

**Published:** 2018-10-27

**Authors:** Liulong Guo, Hongxia Yan, Zhengyan Chen, Qi Liu, Yuanbo Feng, Fan Ding, Yufeng Nie

**Affiliations:** 1 Department of Applied Chemistry, School of Natural and Applied Sciences, Northwestern Polytechnical University, Xi’an 710129, China; guo219823@mail.nwpu.edu.cn (L.G.); chenzhengyan@mail.nwpu.edu.cn (Z.C.); e0344066@u.nus.edu (Q.L.); fengyuanbo244@163.com (Y.F.); df276920255@163.com (F.D.); 2 Department of Applied Mathematics, School of Natural and Applied Sciences, Northwestern Polytechnical University, Xi’an 710129, China; yfnie@nwpu.edu.cn

**Keywords:** reduced graphene oxide, graphene-like WS_2_, bismaleimide, mechanical properties

## Abstract

A novel graphene-based nanocomposite particles (NH_2_-rGO/WS_2_), composed of reduced graphene oxide (rGO) and tungsten disulfide (WS_2_) grafted with active amino groups (NH_2_-rGO/WS_2_), was successfully synthesized by an effective and facile method. NH_2_-rGO/WS_2_ nanoparticles were then used to fabricate new bismaleimide (BMI) composites (NH_2_-rGO/WS_2_/BMI) via a casting method. The results demonstrated that a suitable amount of NH_2_-rGO/WS_2_ nanoparticles significantly improved the mechanical properties of the BMI resin. When the loading of NH_2_-rGO/WS_2_ was only 0.6 wt %, the impact and flexural strength of the composites increased by 91.3% and 62.6%, respectively, compared to the neat BMI resin. Rare studies have reported such tremendous enhancements on the mechanical properties of the BMI resin with trace amounts of fillers. This is attributable to the unique layered structure of NH_2_-rGO/WS_2_ nanoparticles, fine interfacial adhesion, and uniform dispersion of NH_2_-rGO/WS_2_ in the BMI resin. Besides, the thermal gravimetrical analysis (TGA) revealed that the addition of NH_2_-rGO/WS_2_ could also improve the stability of the composites.

## 1. Introduction

Bismaleimide (BMI) resins are a family of thermosetting polymers which have been leading contenders as matrix resins in many cutting-edge fields, especially in aerospace materials sector [1], due to the excellent thermal stability [2], good processability [3], remarkable mechanical properties [4], and prominent dielectric properties [5]. However, the biggest drawback of cured BMI resins is brittleness, which results from the high degree of crosslinking and multiple aromatic structure [6,7]. Consequently, unremitting efforts are made to modify BMI resins to reduce inherent brittleness and achieve elevated properties to satisfy various demands in advanced areas.

A series of studies has revealed that the incorporation of graphene and its derivatives into BMI resins can contribute to significant enhancements of mechanical properties [8]. Graphene oxide (GO) is found in a two-dimensional layered structure that consists of sp^2^-hybridized carbon atoms in the form of hexagonal rings [9]. The presence of functional oxygen groups, like epoxy, hydroxyl, and carboxylic groups, on the basal and edge planes, enlarges the basal spacing of GO and makes the material hydrophilic [10]. GO is a promising substitute for other nanofillers in composites considering the high specific surface area [11], excellent mechanical strength [12], thermal conductivity [13], and electrical properties [14]. In previous reports, functionalized graphene nanosheets with aniline groups on the surfaces (FGN), phosphorous-containing polyhedral oligomeric silsesquioxane-functionalized graphene oxide (P-POSS-GO), and magnetic GO nanosheets (GNS-Fe_3_O_4_@PZM), were synthesized and used to modify BMI resin in trace amounts [15,16,17]. The maximum flexural and impact strength among these studies were 163 MPa and 19.15 kJ/m^2^, respectively. However, the percentage of increment of the flexural and impact strength were dissatisfactory. These results showed that graphene and its derivatives could only slightly improve the mechanical properties of BMI resins so that new particles should be prepared for further modification.

Numerous studies have pointed out that the critical defect of GO lay in the tendency of irreversible agglomeration during processing, due to the large specific surface area and strong van der Waals forces between GO sheets [18]. Recently, two-dimensional transition metal dichalcogenides (TMDs) have captured more attention since they can be fabricated into the graphene-like structure [19], presenting remarkable chemical, physical, and electrical properties [20]. As a member of TMDs, WS_2_ is formed by hexagonally arranged sulfur atoms linked to a tungsten atom and separated by a weak van der Waals gap [21]. With similar morphology and layered structure, GO can be exploited as a buffer layer to form a three-dimensional structure with graphene-like WS_2_. This structure exhibits pure graphene characteristics and prevents the aggregation of GO caused by van der Waals forces [22]. To the best of our knowledge, GO/WS_2_ nanoparticles were widely used as electrocatalysts in electrochemistry [23]; few researchers have incorporated them into matrix resins to elevate mechanical properties.

It is worthy noting that interfacial interaction between graphene-based nanoparticles and the polymeric matrix plays a significant role in achieving the optimal enhancements of the mechanical properties of the composites [24]. One developed method to promote stronger interfacial bonds is to form covalent linkages between graphene-based nanoparticles and the supporting matrix, such as attaching functional groups to the surface of graphene [25], which also improves the dispersibility of graphene-based nanoparticles [26,27]. For example, γ-aminopropyltriethoxysilane (APS) is a kind of coupling agent with active amino groups. When APS is grafted on the surface of graphene-based nanoparticles, the terminal amino groups can covalently bond with the polymeric matrix, reinforcing the interfacial interaction and acquiring better mechanical properties.

In this article, a facile and scalable route to prepare NH_2_-rGO/WS_2_ hybrid nanoparticles from reduced graphene oxide and graphene-like WS_2_ nanosheets was reported. A series of NH_2_-rGO/WS_2_/BMI composites with different loadings of NH_2_-rGO/WS_2_ were prepared, to investigate their structure and effect on properties systematically. Our study revealed that with the incorporation of suitable amounts of NH_2_-rGO/WS_2_ nanoparticles, the BMI composites produced comparatively higher impact and flexural strength than those of other graphene modified BMI resins with trace amount of fillers reported in the previous studies [15,16,17].

## 2. Materials and Methods

### 2.1. Reagents and Materials

WS_2_ powders (<2 μm) were obtained from Macklin Chemistry Co., Ltd. *N*-methyl-2-pyrrolidone (NMP), tetrahydrofuran (THF), ethanol, ammonia water, acetone, and hydrazine hydrate were purchased from Tianjin Tianli Chemical Reagents Co. Ltd. Natural graphite flakes (325 mesh) were purchased from Qingdao Hensen Graphite Co. Ltd. The graphene oxide (GO) nanosheets were fabricated from natural graphite flakes via a modified Hummers’ method [28]. γ-Aminopropyltriethoxysilane (APS) was provided by Jingzhou Jianghan Fine Chemical Co. Ltd. BMI was provided by Rongchang Ning research group at Northwestern Polytechnical University. Diallyl Bisphenol A (DBA) and 4,4′-bismaleimidodiphenylmethane (BDM) were purchased from Sigma-Aldrich. All reagents were of analytical grade, and used as received without further purification.

### 2.2. Experimental Section

#### 2.2.1. Synthesis of Graphene Oxide/WS_2_ Nanosheets (GO/WS_2_)

Nanostructured WS_2_ was prepared from commercial bulk WS_2_ by a mechanochemical treatment method [29]. In a typical synthesis process, 0.5 g of WS_2_ and 5.0 g of NaCl, with a ball feed ratio of 1:7, were added to the agate grinding bowl of a planetary ball mill, grinding for 2 h at a rotation rate of 560 rpm. The resulted solid was washed by deionized water repeatedly to remove NaCl, and was dried at 100 °C under vacuum for 8 h to obtain the exfoliated WS_2_ nanosheets (exf-WS_2_). Then exf-WS_2_ was dissolved in NMP and stirred under ultrasound to disperse thoroughly. The obtained solution was centrifugated for 1.5 h at the rotation rate of 4000 rpm, after which the upper half of its volume was collected.

Subsequently, 500 mL of GO aqueous suspension was rotary-evaporated and dispersed in NMP under ultrasound for 30 min. Then exf-WS_2_ was added and sonicated for 2 h. The hybrid solution was kept at 25 °C for 4 days, then 1 mL of concentrated hydrochloric acid was added into the solution with stirring. The mixture was further reacted at 80 °C for 1 day, after which it was cooled down to normal temperature and was transferred to absolute ethanol, filtered, and washed for several times. Finally, the product was dried at 60 °C in vacuum oven for more than 6 h.

#### 2.2.2. Preparation of NH_2_-rGO/WS_2_ Hybrid Nanoparticles

The experimental procedures can be concluded as two steps: (1) the reaction to graft APS on the surface of GO/WS_2_, and (2) the reducing reaction to obtain NH_2_-rGO/WS_2_ using hydrazine hydrate, and the reduction of GO is aimed at promoting the intercalation with WS_2_ to prevent the agglomeration of nanoparticles.

In the first step, GO/WS_2_ was dispersed in the mixture solution of 350 mL of ethanol and 16 mL of deionized water. After 30 min ultrasonication, 5 mL of APS, dissolved in 50 mL of ethanol and 4 mL of deionized water, was dropped into GO/WS_2_ dispersion via a constant-pressure funnel. The dispersion was heated to 78 °C, and maintained for 4 h. Following washing with ethanol repeatedly, NH_2_-GO/WS_2_ nanoparticles were obtained after drying in a vacuum oven at 60 °C for 12 h.

**Scheme 1:**
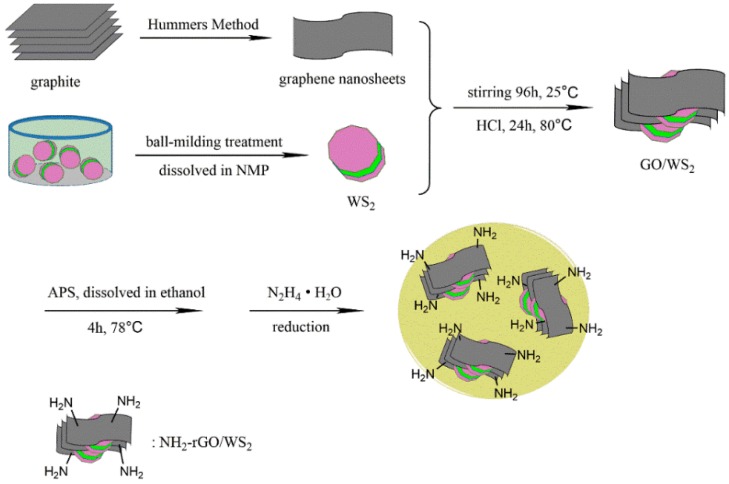
The synthetic route of NH_2_-rGO/WS_2._

In the second step, NH_2_-GO/WS_2_ was dispersed in 450 mL of deionized water under ultrasound for 30 min, and was transferred to a 500 mL three-necked flask holding a mechanical stirrer and reflux-condenser. Then, 10 mL of ammonia water and 4 mL of hydrazine hydrate were added. The mixture was heated in oil bath at 98 °C for 6 h. The product, abbreviated as NH_2_-rGO/WS_2_, was washed with deionized water several times, and dried in the vacuum oven at 60 °C for 8 h. The synthetic route of NH_2_-rGO/WS_2_ is shown in Scheme 1.

#### 2.2.3. Preparation of NH_2_-rGO/WS_2_/BMI Composites

The NH_2_-rGO/WS_2_/BMI composites were fabricated using a casting method. With a mass ratio of 3:4, DBA and BDM were blended in a beaker to prepare the pre-polymer, maintaining the temperature at 140 °C until thoroughly melted. Then, a suitable amount of NH_2_-rGO/WS_2_ was added to the pre-polymer and stirred for about 30 min to disperse homogeneously. Subsequently, the mixture was carefully poured into a pre-heated mold coated with release agent and degassed in the vacuum oven at 150 °C for 1 h. Afterwards, the mixture was cured and post-cured following the schedule of 150 °C/2 h, 180 °C/2 h, 220 °C/3 h and 250 °C/4h. Finally, the mold cooled down to room temperature, and was demolded to obtain the samples of NH_2_-rGO/WS_2_/BMI composites.

### 2.3. Characterization

Fourier transform infrared spectra (FT-IR) of the samples were tested from 4000 to 400 cm^−1^ with a Nicolet FT-IR 5700 spectrometer (USA), of which the resolution was 2 cm^−1^. The X-ray diffraction (XRD) patterns of the samples were determined with a Bruker D8 ADVANCE X-ray diffractometer emitting Cu Kα radiation (λ = 0.15405 nm). X-ray photoelectron spectroscopy (XPS, Thermal Scientific K-Alpha XPS spectrometer) was adopted to investigate the elemental composition on the surface and analyze the valence state of the elements. The transmission electron microscopy (TEM) images were recorded on a JEOL JEM-200CX instrument.

Impact strength was measured based on GB/T 2567-2008 (Chinese Standard). Samples were divided into strips of (80 ± 0.2) × (10 ± 0.2) × (4 ± 0.2) mm^3^ using a cutting machine. The impact speed towards the center of samples was 2.9 m/s, controlling the extreme deviation under ±10%. Flexural strength was determined based on GB/T 2567-2008 (Chinese Standard). Samples were cut into strips of (80 ± 0.2) × (15 ± 0.2) × (4 ± 0.2) mm^3^. Scanning electron micrographs (SEM) were carried out on a Hitachi S-570 instrument (Tokyo, Japan) to observe the surface morphology of impact fracture. Thermal gravimetrical analysis (TGA) were obtained on Perkin Elmer TGA-7 (Waltham, MA, USA) at a heating rate of 10 °C min^−1^, from 50 to 800 °C, in an argon atmosphere. Calorimetry studies were carried out on a TA Instruments DSC 2920 (TA Instrument, New Castle, DE, USA) under ultrahigh-purity nitrogen as inert atmosphere, from 30 to 300 °C, with a heating rate of 10 °C/min. Thermal conductivity of samples were measured at room temperature by a Hot Disk instrument (AB Co, Uppsala, Sweden).

## 3. Results and Discussion

### 3.1. Characterization of NH_2_-rGO/WS_2_ Nanoparticles

**Figure 1:**
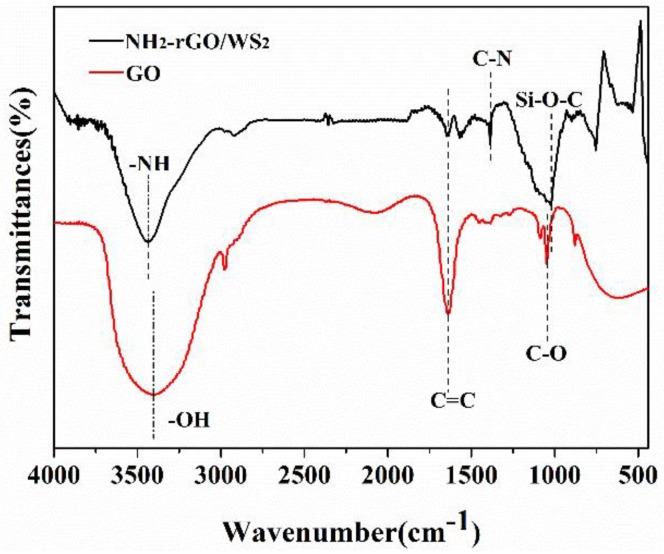
FT-IR spectra of as-prepared GO and NH_2_-rGO/WS_2_ nanoparticles.

The FT-IR spectra of GO and NH_2_-rGO/WS_2_ are shown in Figure 1. In the spectrum of GO, the adsorption band at 3423 cm^−1^ is assigned to –OH, indicating that GO has been oxidized. The typical bands at 1643 and 1040 cm^−1^ are ascribed to C=C and C–O vibrations, respectively. By contrast, –OH and C–O vibrations at 3423 and 1040 cm^−1^ disappear in the spectrum of NH_2_-rGO/WS_2_, while the peak of C=C still exists, proving the reduction of GO. In addition, the adsorptions at 3450, 1380, and 1080 cm^−1^ are attributed to –NH_2_, C–N, and Si–O–C, suggesting that APS has been successfully grafted to the surface of rGO/WS_2_.

**Figure 2:**
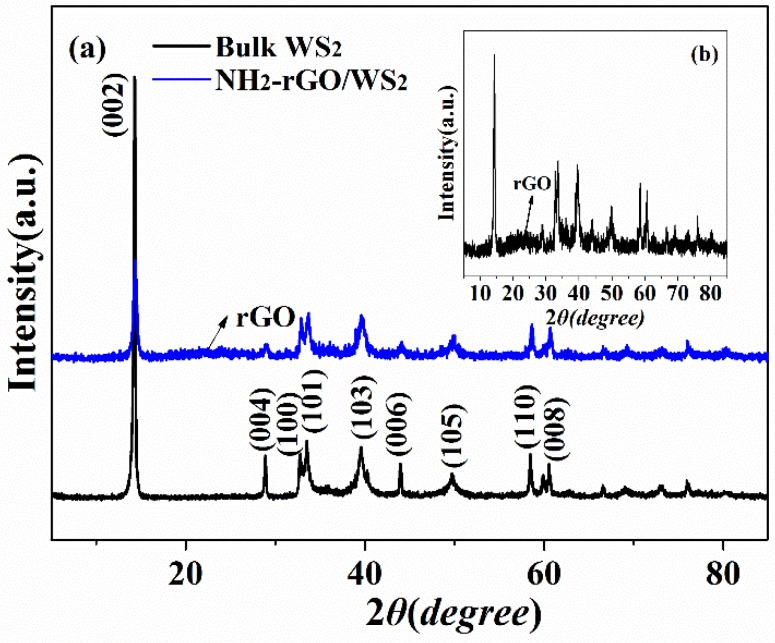
XRD patterns of bulk WS_2_ and NH_2_-rGO/WS_2_ nanosheets.

The crystallographic phases of bulk WS_2_ and NH_2_-rGO/WS_2_ was investigated by XRD spectra, which is shown in Figure 2. The XRD spectrum of bulk WS_2_ demonstrates highly crystalline hexagonal structure without any impurities. The strong (002) peak located at 2θ = 14.3° corresponded to a d-spacing of 0.62 nm, according to the Bragg equation, indicating a well-stacked layered structure along the c axis [30]. The XRD pattern of the bulk WS_2_ shows sharper and more intense peaks as compared to NH_2_-rGO/WS_2_ nanoparticles, indicating both smaller lattice size and fewer layers of NH_2_-rGO/WS_2_ along the c axis. In the magnification spectrum of XRD, all the diffraction peaks of NH_2_-rGO/WS_2_ can be observed, as well as a new diffraction peak of rGO at 2θ ≈ 24.0°, confirming the triumphant synthesis of NH_2_-rGO/WS_2_. The existence of rGO enables further decrease of WS_2_ layers in NH_2_-rGO/WS_2_.

**Figure 3:**
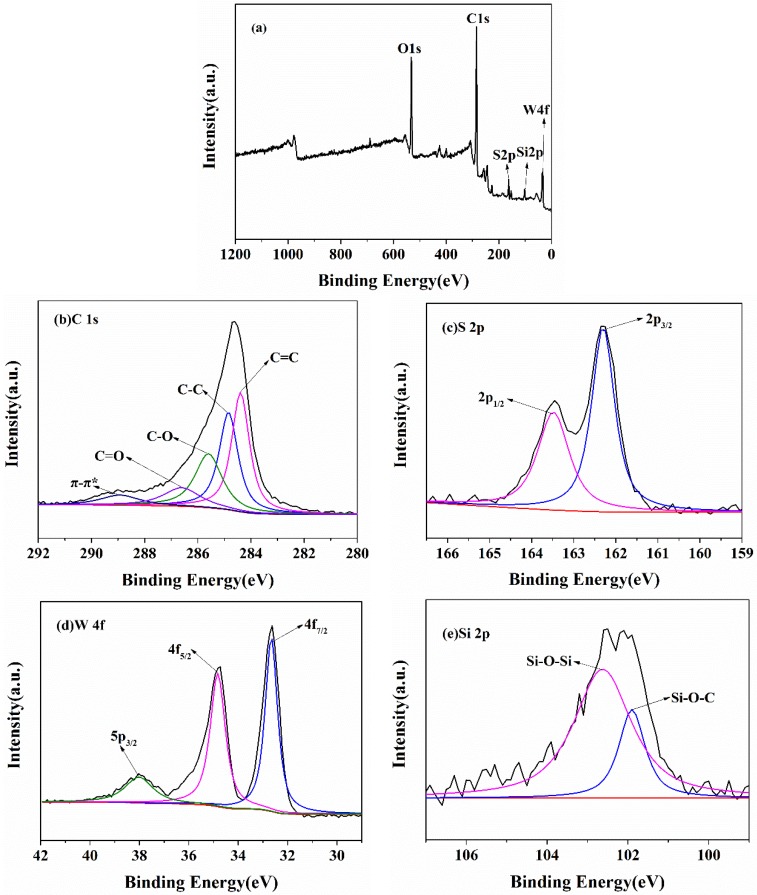
XPS spectra of (**a**) NH_2_-rGO/WS_2_, (**b**) C 1s, (**c**) S 2p, (**d**) W 4f and (**e**) Si 2p of NH_2_-rGO/WS_2_.

XPS is employed to characterize the chemical composition and electronic structure of NH_2_-rGO/WS_2_ nanoparticles. Figure 3a shows the full-band XPS spectrum of NH_2_-rGO/WS_2_. Five obvious peaks can be observed at the binding energies of 285.0, 532.0, 102.0, 162.0, and 34.0 eV, which are ascribed to C 1s, O 1s, Si 2p, and W 4f, respectively. S atom and W atom come from WS_2_, while Si atom derives from APS, which proves APS has been grafted to the surface of rGO/WS_2_. There are five peaks (Figure 3b) at the binding energies of 284.4, 284.9, 285.8, 286.7, and 288.9 eV in the spectrum of C 1s, attributed to sp^2^-hybridized C=C, sp^3^-hybridized C–C, carbon in C–O bond, carbon in C=O bond, and a π–π* peak [7]. The S 2p peak-fitting (Figure 3c) clearly reveals two resolved peaks at 162.3 eV and 163.5 eV, assigned to S 2p_3/2_ and S 2p_1/2_. The W 4f peak-fitting spectrum (Figure 3d) exhibits three peaks at 32.0, 34.1, and 37.3 eV, which are indexed to W 4f_7/2_, W 4f_5/2_, and W 5p_3/2_, respectively [22]. In the spectrum of Si 2p (Figure 3e), two peaks at 101.9 eV and 102.6 eV correspond to Si–O–C bond and Si–O–Si bond [31]. From the XPS spectra above, active –NH_2_ functional groups are successfully grafted on the surface of rGO/WS_2_.

**Figure 4:**
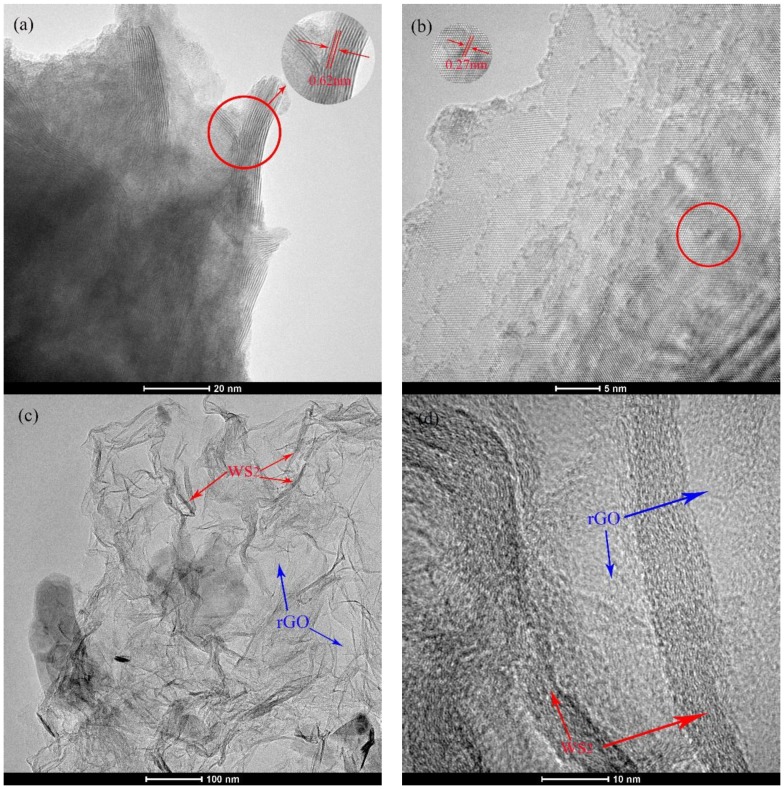
TEM and HRTEM images of (**a**,**b**) bulk WS_2_; (**c**,**d**) WS_2_/rGO.

To better verify the microstructures of WS_2_ and NH_2_-rGO/WS_2_, TEM and HRTEM studies were carried out. As is shown in Figure 4a,b, WS_2_ demonstrates a well-layered structure with d(002) = 0.62 nm and periodic arrays of (100) planes with a W-S lattice spacing of 0.27 nm, which coincides with the result of XRD analysis and the hexagonal lattice of the WS_2_ phase. It is labeled in Figure 4c that WS_2_ has been anchored on GO nanosheets homogeneously, indicating the successful combination of WS_2_ and GO nanosheets. The HRTEM image in Figure 4d explicitly exhibits that WS_2_ and GO nanosheets appear alternatively, forming a multilayered hybrid structure.

### 3.2. Mechanical Properties of NH_2_-rGO/WS_2_/BMI Composites

**Figure 5:**
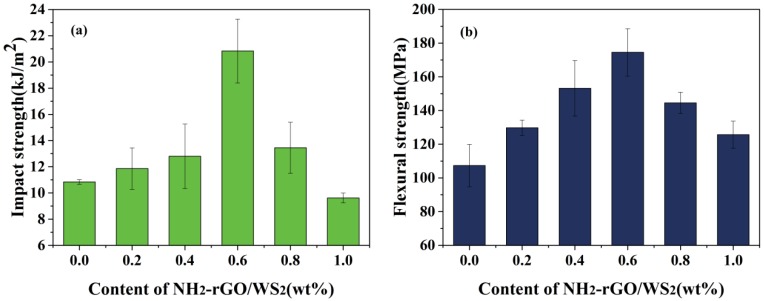
Impact strength (**a**) and flexural strength (**b**) of BMI composites with different contents of NH_2_-rGO/WS_2_ fillers.

In this work, it is investigated that the impact and flexural strength of NH_2_-rGO/WS_2_/BMI composites depend on the content of NH_2_-rGO/WS_2_ (from 0 to 1.0 wt %), shown in Figure 5a,b. An appropriate amount of NH_2_-rGO/WS_2_ can significantly improve the toughness and the ability to resist static bending moments. The impact and flexural strength increase continuously with the addition of NH_2_-rGO/WS_2_, peaking at 20.8 kJ/m^2^ and 174 MPa when adding 0.6 wt % NH_2_-rGO/WS_2_, before taking a downward trend. Compared with the pure BMI resin (10.8 kJ/m^2^ and 107 MPa), the impact and flexural strength of NH_2_-rGO/WS_2_/BMI increase by 91.3% and 62.6%, respectively. Rarely have previous studies achieved such remarkable improvements in the flexural and impact strength of graphene-based nanoparticle-modified BMI or other resins with trace amounts of fillers. Some previous works have been listed in Table 1; to the best of our knowledge, the comparatively optimal flexural and impact strength of graphene-based nanoparticle-modified BMI, or other resins among these studies, were 163 MPa and 19.15 kJ/m^2^, respectively [4,15,16,17,32,33,34]. Thus, the results of the present work are comparable to that of previous works, indicating the NH_2_-rGO/WS_2_ nanoparticles play a vital role in elevating the mechanical properties of the BMI resin.

Two reasons account for the enhancement of mechanical properties of NH_2_-rGO/WS_2_/BMI at a low content of fillers: (1) the fine dispersion of WS_2_ and GO in BMI resin by sonication results in enhanced interactions between nanofillers and polymer matrix. Thus, when the distribution of the nanofiller is more homogenous without agglomeration, the interface can transfer load more effectively [35]; (2) there exists plenty of amine groups that can react with C=C bonds of BMI resin on the edge of NH_2_-rGO/WS_2_ nanoparticles, so that the bonding strength between fillers and BMI resin is reinforced [36]. However, the mechanical properties of the composites are deteriorated as the content of NH_2_-rGO/WS_2_ surpasses 0.6 wt %. This phenomenon can be explained by the fact that excessive NH_2_-rGO/WS_2_ nanoparticles cannot be well dispersed in the BMI matrix. Instead, they are easy to agglomerate to cluster, contributing to stress concentration and weak interface adhesion [37]. Besides, at the procedure of curing, high concentration of NH_2_-rGO/WS_2_ thickens the BMI resin, not only impeding the volatilization of acetone in resin matrix, but also causing the generation of defects in the NH_2_-rGO/WS_2_/BMI composites [38]. Thus, only when the content of NH_2_-rGO/WS_2_ fillers is suitably added can the composites preform excellent mechanical properties.

**Figure 6:**
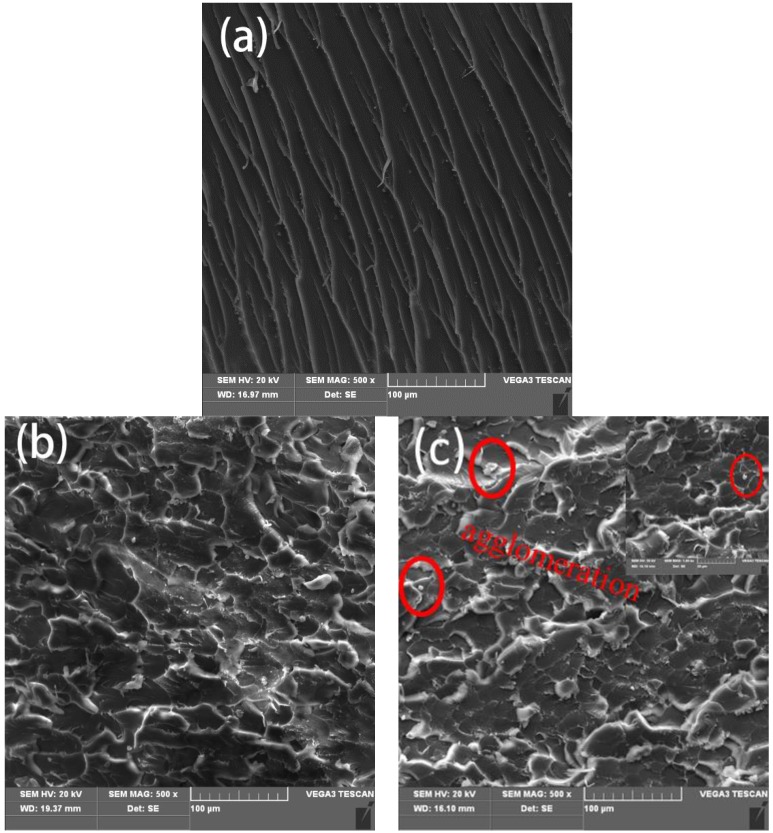
SEM images of the fracture surfaces of (**a**) the neat BMI (**b**) the composite with 0.6 wt % NH_2_-rGO/WS_2_ and (**c**) the composite with 1.0 wt % NH_2_-rGO/WS_2_.

To obtain more information concerning the toughening mechanism of NH_2_-rGO/WS_2_ in BMI matrix, the impact fracture surfaces were detected by SEM. Figure 6a–c demonstrate the impact fracture surfaces of pure BMI resin, NH_2_-rGO/WS_2_/BMI resin with 0.6 wt % fillers and NH_2_-rGO/WS_2_/BMI resin with 1.0 wt % fillers, respectively. Typical characteristics of brittle failure are explicitly presented in the fracture surface of neat BMI resin (Figure 6a). The fracture surface is relatively flat and smooth without interrupted crack propagation path, resulting in poor energy consumption during fracture. However, BMI composite containing 0.6 wt % NH_2_-rGO/WS_2_, shown in Figure 6b, has coarser surface with abundant ridges, ravine patterns, and dimples, presenting typical characteristics of ductile fracture. It also can be observed that a few NH_2_-rGO/WS_2_ nanoparticles appear around the dimples, implying that these dimples originated from NH_2_-rGO/WS_2_ nanoparticles [4]. Therefore, the highly dispersed NH_2_-rGO/WS_2_ contributes to the occurrence of dimples and dominant plastic deformation of BMI matrix, impeding further crack propagation and consuming much more energy, whereas the agglomerations of fillers can be seen on the fracture surface of the 1.0 wt % NH_2_-rGO/WS_2_/BMI composites (Figure 6c), leading to the deteriorated mechanical properties of the BMI composites.

### 3.3. Thermal Properties of NH_2_-rGO/WS_2_/BMI Composites

**Figure 7:**
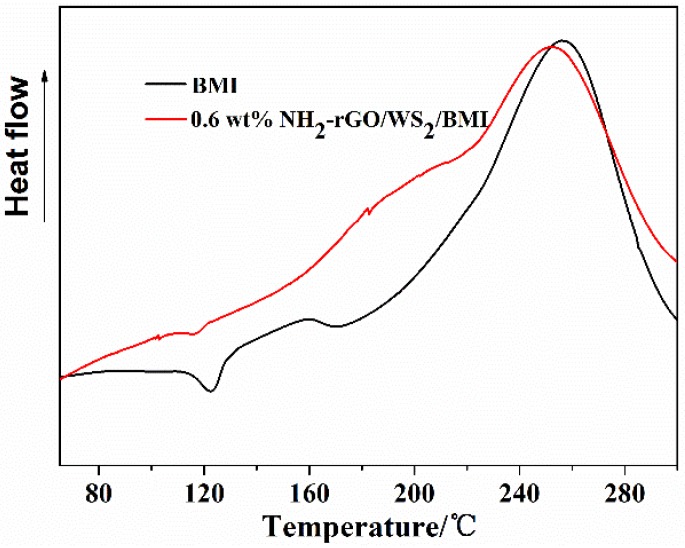
DSC curves of the neat BMI resin and 0.6 wt % NH_2_-rGO/WS_2_/BMI resin with a heating rate of 10 °C/min.

**Scheme 2:**
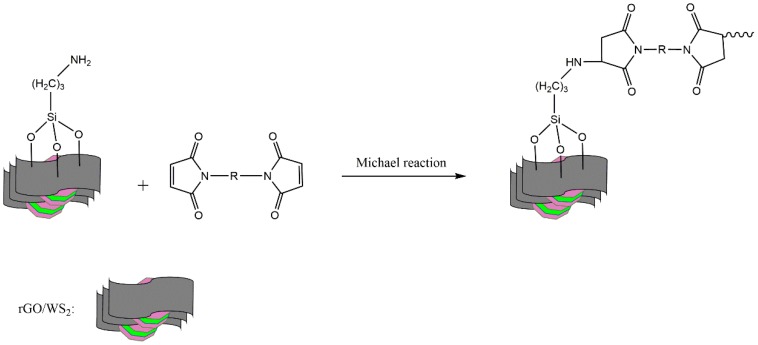
The mechanism of Michael reaction of the amino groups on the NH_2_-rGO/WS_2_ particles to maleimide groups.

The curing behavior of a thermosetting system determines the structure, as well as the properties, of the cured resin. Figure 7 gives the DSC curves of the neat BMI resin and 0.6 wt % NH_2_-rGO/WS_2_/BMI composite. The neat BMI resin presents a sharp and strong exothermic peak at 257.4 °C, due to homopolymerization of BMI. By contrast, the exothermic peak of 0.6 wt % NH_2_-rGO/WS_2_/BMI composite shifts to the left with a peak temperature around 252.5 °C, and becomes broader. This is because that the amino groups on the edges of NH_2_-rGO/WS_2_ particles can react with imide rings through the Michael reaction [39]. The detailed reaction mechanism is shown in Scheme 2. It is notable that NH_2_-rGO/WS_2_/BMI resin shows better processability, since the whole exothermic peak of NH_2_-rGO/WS_2_/BMI composite appears at a lower temperature than that of the neat BMI resin.

**Figure 8:**
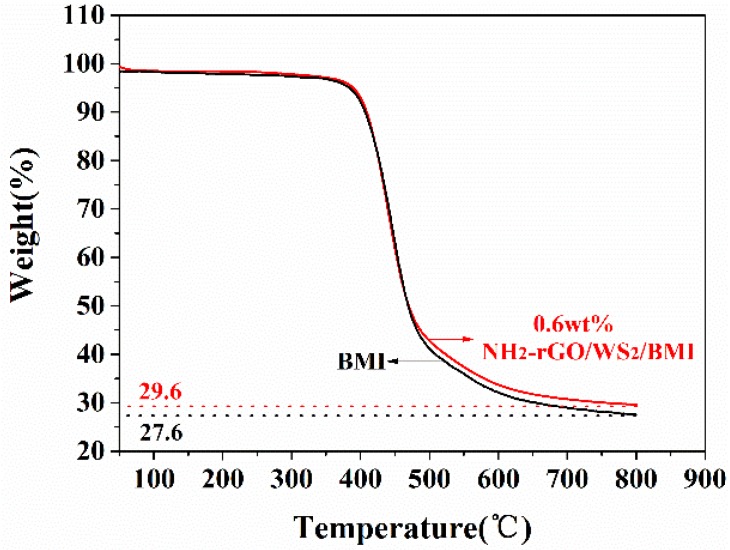
TGA curves of neat BMI and 0.6 wt % NH_2_-rGO/WS_2_/BMI composite.

Figure 8 shows the overlay curves of weight loss of the cured neat BMI resin and BMI composite with 0.6 wt % of NH_2_-rGO/WS_2_ nanoparticles. As expected, BMI composite with 0.6 wt % of NH_2_-rGO/WS_2_ nanoparticles exhibit slightly higher decomposition temperature and a better thermal stability than the neat BMI resin. The char yield of the neat BMI resin at 800 °C is 27.6%. It is exciting that the addition of 0.6 wt % of NH_2_-rGO/WS_2_ nanoparticles efficiently raises the char yield of the BMI composite at 800 °C to 29.6%, with an increase of 7.25%. The result indicates that NH_2_-rGO/WS_2_ fillers can improve the thermal property of the neat BMI resin. This can be explained by the barrier effect of modified GO nanosheets, uniform dispersion of NH_2_-rGO/WS_2_ fillers, and strong interfacial bonding between NH_2_-rGO/WS_2_ nanoparticles and BMI matrix [40].

## 4. Conclusions

In this study, reduced graphene oxide and WS_2_ nanoparticles with active amino groups (NH_2_-rGO/WS_2_) were synthesized. BMI composites incorporated with NH_2_-rGO/WS_2_ nanoparticles at different loadings were prepared. The impacts of NH_2_-rGO/WS_2_ nanoparticles on the mechanical and thermal properties of the BMI resin were investigated and compared. Various characterizations, including FTIR, XPS, XRD, and TEM, demonstrated the successful preparation of NH_2_-rGO/WS_2_. Owing to the unique layered structure and the chemical interaction between amino groups on the surface of rGO/WS_2_ nanoparticles and C=C bonds of BMI matrix, the dispersion and the interfacial adhesion were significantly enhanced, which further improved the mechanical properties of NH_2_-rGO/WS_2_/BMI composites considerably. The maximum increment of the impact and flexural strength of the composites (20.8 kJ/m^2^ and 174 MPa) were 91.3% and 62.6%, respectively, compared with the neat BMI resin (10.8 kJ/m^2^ and 107 MPa), at the loading of 0.6 wt % NH_2_-rGO/WS_2_ nanoparticles. In contrast with the optimal impact and flexural strength of graphene-modified BMI resins reported (19.15 kJ/m^2^ and 163 MPa), trace amounts of NH_2_-rGO/WS_2_ nanoparticles promoted almost the best enhancements on mechanical properties of the BMI resin. In addition, the system showed a slight rise on the thermostability of the NH_2_-rGO/WS_2_/BMI composites.

## Figures and Tables

**Table 1:** Mechanical properties of bismaleimide and other polymer composite with different fillers.

Polymers	Fillers	Optimal Loading	Remarks	Reference
BMI	NH_2_-rGO/WS_2_	0.6 wt %	The flexural and impact strength of the composites increased by 62.6% and 91.3%, respectively, compared to the neat BMI	This paper
BMI	GO	0.15 wt %	The flexural and impact strength of the composites increased by 19.82% and 32.48%, respectively, compared to the neat BMI	[4]
BMI	MAH-GO	0.1 wt %	The flexural and impact strength of the composites increased by 37.48% and 77.28%, respectively, compared to the neat BMI	[4]
BMI	MPTS-GO	-	The tensile and impact strength of the composites increased by 22.17% and 66.64%, respectively, compared to the neat BMI	[8]
BMI	GNS-Fe_3_O_4_@PZM	0.4 wt %	The flexural and impact strength of the composites increased by 31.3% and 61.3%, respectively, compared to the neat BMI	[16]
BMI	P-POSS-GO	0.8 wt %	The flexural and impact strength of the composites increased by 24.6% and 100.8%, respectively, compared to the neat BMI	[17]
CE/BMI	FGONs	1.0 wt %	The flexural and impact strengths reached optimum values of 110 MPa and 10.98 kJ m^−2^	[15]
Epoxy	WS_2_-PEI	0.25 wt %	The fracture toughness increased by 82.98% and flexural strength increased by 65%, compared to the neat EP	[32]
Epoxy	IF-WS_2_	0.5 wt %	The composite exhibited high shear and peel strength	[33]
Nylon 12	IF-WS_2_	2 wt %	The tensile strength and bending strength of the composites increased by 27% and 28%, respectively	[34]

BMI, bismaleimide; MAH-GO, maleic anhydride-functionalized GO; MPTS-GO, γ-methacryloxypropyltrimethoxysilane-functionalized GO; GNS-Fe_3_O_4_@PZM, reduced graphene oxide-Fe_3_O_4_@polyphosphazene; P-POSS-GO, phosphorus-containing polyhedral oligomeric silsesquioxane-functionalized Graphene oxide; CE, cyanate ester; FGONs,γ-(2,3-epoxypropoxy)propyltrimethoxysilane-functionalized graphene oxide nanoflakes; WS_2_-PEI, branched polyethyleneimine-functionalized WS_2_; IF-WS_2_, fullerene-like WS_2_.
